# Biotechnological applications of archaeal enzymes from extreme environments

**DOI:** 10.1186/s40659-018-0186-3

**Published:** 2018-10-05

**Authors:** Ma. Ángeles Cabrera, Jenny M. Blamey

**Affiliations:** 1 Fundación Científica y Cultural Biociencia, José Domingo Cañas, 2280 Santiago, Chile; 2 Facultad de Química y Biología, Universidad de Santiago de Chile, Avenida Libertador Bernardo O´Higgins, 3363 Santiago, Chile

**Keywords:** Antarctica, Archaea, Extremozymes, Biocatalysis

## Abstract

To date, many industrial processes are performed using chemical compounds, which are harmful to nature. An alternative to overcome this problem is biocatalysis, which uses whole cells or enzymes to carry out chemical reactions in an environmentally friendly manner. Enzymes can be used as biocatalyst in food and feed, pharmaceutical, textile, detergent and beverage industries, among others. Since industrial processes require harsh reaction conditions to be performed, these enzymes must possess several characteristics that make them suitable for this purpose. Currently the best option is to use enzymes from extremophilic microorganisms, particularly archaea because of their special characteristics, such as stability to elevated temperatures, extremes of pH, organic solvents, and high ionic strength. Extremozymes, are being used in biotechnological industry and improved through modern technologies, such as protein engineering for best performance. Despite the wide distribution of archaea, exist only few reports about these microorganisms isolated from Antarctica and very little is known about thermophilic or hyperthermophilic archaeal enzymes particularly from Antarctica. This review summarizes current knowledge of archaeal enzymes with biotechnological applications, including two extremozymes from Antarctic archaea with potential industrial use, which are being studied in our laboratory. Both enzymes have been discovered through conventional screening and genome sequencing, respectively.

## Background

Biotechnology is present everywhere and it has had a great impact on several industrial sectors, such as fine chemical and pharmaceutical industry, textile, and detergent industries, biofuel generation, bioremediation, among others. Generally, industrial processes use chemical compounds as catalysts, generating toxic byproducts [1]. Biocatalysis has emerged as an alternative process using enzymes or cells as biocatalysts, which are more selective, efficient and environmentally friendly [2]. Mesophilic enzymes have been used as biocatalysts but they have low stability at high temperature or extremes pH. For this reason, there is a considerable demand for more stable enzymes. One approach to overcome this need is to search for new enzymes within extremophilic microorganisms. Extremophiles are organisms that are able to thrive at extreme environmental conditions (temperature, pressure, salinity, dryness, radiation, pH or concentrations of heavy metals) (see Table 1). Most of the extremophiles belong to the Archaea domain. These microorganisms and their enzymes have unique characteristics [3, 4]. Archaea exist in a broad range of habitats, but there are a few reports of thermophilic or hyperthermophilic archaea from Antarctica [5]. This is an extreme continent not only composed of cold environments but also of geothermal sites, such as fumaroles, hot springs, hydrothermal vents, and volcanoes. These sites are suitable for the development of these microorganisms.

**Table 1:** Classification of extremophiles and examples of their habitats. Adapted from [6, 7]

Type	Growth characteristics	Habitat
Acidophiles	Low pH (< 2)	Hot sulfur springs, waste treatment plants, and mine drainage
Alkaliphiles	High pH (> 10)	Soda lakes, alkaline hot springs, deserts, and mine waste
Halophiles	High concentration of salt (2–5 M NaCl)	Salt lakes, coastal lagoons, and saline soils
Metallophiles	High concentration of heavy metals (Cu, Cd, As, Zn)	Deep-sea or terrestrial hydrothermal sources and metal-processing factories
Piezophiles or barophiles	High hydrostatic pressure (40–130 MPa)	Ocean floor and deep-sea hot vents
Psychrophiles	Low temperature (< 15 °C)	Arctic and Antarctic soils and waters, alpine soils, deep ocean water, and glaciers
Radiophiles	High levels of ionizing radiation (> 25 kGy); 5 Gy is lethal for humans	Terrestrial surfaces, upper layers of the sea, and nuclear waste
Thermophiles	High temperature Thermophiles (60–80 °C) Hyperthermophiles (> 80 °C)	Deep-sea or shallow hydrothermal vents, hot springs, geysers, volcanoes, coal refuse piles, and industrial hot water systems
Xerophiles	Low water activity (aw ≤ 0.8)	Deserts and salt beds

Extremophilic archaea that live under extreme conditions have developed enzymes with unique structure–function properties. These enzymes, known as extremozymes, have an increased stability at high temperatures, extreme pH, in the presence of organic solvents and heavy metals and against proteolytic attack. For this reason, they are able to withstand harsh conditions during industrial processes and can be used in a diversity of biotechnological applications (see Table 2). To date, there are a variety of archaeal extremozymes, which are used as biocatalysts in different industrial sectors [8]. In this work archaeal extremozymes with biotechnological applications and potential use will be reviewed, including an Antarctic extremozyme that has being developed in our laboratory.

**Table 2:** Characteristics of archaeal extremozymes and their applications. Adapted from [7–10]

Type	Characteristics of the enzymes	Enzymes	Applications
Acidophiles	Prevalence of acidic amino acids on the surface	Amylases, glucoamylases, xylanases, cellulases, proteases	Biofuel production, food, mining, starch processing, desulfurization of coal
Alkaliphiles	Prevalence of basic amino acids on the surface, high pI values	Proteases, cellulases amylases	Detergents, food and feed, beer and paper industry
Halophiles	Relatively large number of acidic amino acids on the surface, smaller hydrophobic amino acids and salt-dependent folding	Proteases, dehydrogenases	Peptide synthesis, biocatalysis in organic media
Psychrophiles	Smaller number of disulfide bonds, hydrogen bonds and salt bridges. Decrease in hydrophobic property, lower thermal stability, increased flexibility and specific activity	Proteases, amylases, cellulases, lipases	Laundry, detergents, textiles
Thermophiles	Increase in ionic interactions, increased hydrophobicity, packing, number of disulfide bonds, salt-bridging, surface charges, shortening of surface loop, stabilization of loops by interaction with metal ions, reduction in unstable amino acids at high temperatures	Proteases, lipases, glucoamylases, glucosidases, amylases, pullulanases, cellulases, xylanases, esterases, DNA polymerases, dehydrogenases	Detergents, food and feed, starch, cellulose, textiles, paper bleaching, molecular biology, oxidation reactions, fine chemicals and pharmaceuticals

## Proteolytic enzymes (EC 3.4.x.x)

Proteases catalyze hydrolysis of proteins into smaller peptides or free amino acids. They are generally classified in two groups: endopeptidases (proteases that cleave peptide bonds within the protein) and exopeptidases (proteases that cleave off amino acids from the ends of the protein). Based on the mechanism of catalysis they are classified in serine proteases (EC 3.4.21.x), cysteine or thiol proteases (EC 3.4.22.x), aspartic, carboxyl or acidic proteases (EC 3.4.23.x), glutamic proteases (EC 3.4.23.x), threonine proteases (EC 3.4.25.x), and metalloproteases (EC 3.4.24.x) [11]. Proteases are of great interest because of their versatile characteristics and different applications in industrial sectors. These enzymes represent a large percentage of the global enzyme market [12, 13]. Most proteases from extremophiles belong to the serine type and many of them come from hyperthermophilic archaea belonging to the genera *Pyrococcus* [14], *Thermococcus* [15], *Desulfurococcus* [16], *Pyrobaculum* [17], *Staphylothermus* [18], and from the thermoacidophilic archaeon *Sulfolobus* [19] (see Table 3).

**Table 3:** Proteolytic enzymes from archaea

Type of protease	Organism	Type of enzyme	Enzyme properties			References
			Optimal temperature (°C)	Optimal pH	Thermostability	
Serine proteases	*Desulforococcus* strain Tok_12_S_1_	Native	95	7.2	70–90 min, 95 °C	[16]
	*Pyrobaculum aerophilum* IM2	Native	100–130	Neutral-alkaline	Not reported	[17]
	*Staphylothermus marinus*	Native	90	9	Not reported	[18]
	*Thermococcus kodakaraensis*	Recombinant	100	7–11.5	100 min, 100 °C	[15]
	*Haloferax lucentensis* VKMM 007	Native	60	8.0	> 30 min, 60 °C	[20]
Thiol protease	*Pyrococcus horikoshi* OT3	Recombinant	80	8.5	Not reported	[14]
Acidic protease	*Sulfolobus solfataricus* P2	Native	70	2	Not reported	[19]

In addition, there are also proteases derived from halophilic archaea belonging to the genera *Haloferax* [20], *Halobacterium* [21, 22], *Natrinema* [23], and *Natronomonas* [24]. These enzymes are alkaline proteases, they work at elevated pH and some of them are stable at high temperatures or in organic solvents. For example, a protease from *Haloferax lucentensis VKMM 007* showed maximal activity at 60 °C at pH 8 and it remains active in the presence of various polar and non-polar solvents, surfactants and reducing agents [20].

### Biotechnological applications of proteases

In food and feed industry they are used to degrade complex proteins, predigest baby foods or soft meat. Since the latter process is carried out at 40–60 °C, thermostable proteases are mainly required for this purpose [10]. In detergent industry they are used as additives in household laundry detergents to remove proteinaceous stains. In this industry, proteases have also been shown to resist denaturation by detergents and alkaline conditions. Thus, alkaline proteases from halophilic archaea are ideal for this purpose [25]. In molecular biology they are used to remove proteinaceous contaminants of DNA in PCR prior to amplification. Therefore, thermostability to function in PCR is absolutely required. In peptide synthesis the process is carried out in low water/nonaqueous environments and peptides are used as precursors of sweeteners, such as aspartame. Thus, alkaline proteases resistant to organic solvents are required [10]. Proteases can also help to reduce time during dough fermentation in bread industry and to modify mixtures containing high gluten content, through partial hydrolysis of the blend, making it soft and easy to pull and knead [25].

## Esterases (EC 3.1.1.1) and lipases (EC 3.1.1.3)

Esterases and lipases are widely used as biocatalysts in biotechnology. Esterases (EC 3.1.1.1) hydrolyze water-soluble short acyl chain esters. On the other hand, lipases (EC 3.1.1.3) catalyze the hydrolysis of long-chain acylglycerols into glycerol and fatty acids. These enzymes display much broader substrate specificity than esterases. Esterases and lipases possess regio-, chemo-, and enantioselectivity and are stable in organic solvents. Thus, both types of enzymes are widely used in industrial processes performed in organic solvents [26, 27]. Many hyper/thermophilic esterases and lipases come from archaea belonging to the genera *Pyrococcus* [28], *Pyrobaculum* [29], *Sulfolobus* [30], *Aeropyrum* [31], and *Archaeoglobus* [32, 33]. These enzymes have also been reported from halophilic archaea belonging to the genera *Haloarcula* [34] and *Halococcus* [35] (see Table 4).

**Table 4:** Esterases and lipases from hyper/thermophilic archaea

Organism	Enzyme	Type of enzyme	Enzyme properties			References
			Optimal temperature (°C)	Optimal pH	Thermostability	
*Pyrococcus furiosus*	Lipase	Recombinant	80	7	6 h, 75 °C	[28]
*Pyrobaculum* sp. 1860	Esterase	Recombinant	80	9	6 h, 90 °C	[29]
*Sulfolobus tokodaii* strain 7	Esterase	Recombinant	70	7.5–8.0	40 min, 85 °C	[30]
*Archaeoglobus fulgidus*	Lipase	Recombinant	90	10	Not reported	[33]

### Biotechnological applications of esterases and lipases

Esterases and lipases are used in fine chemicals production (chemicals produced with purity higher than 90%) and pharmaceutical industry. They are used to improve the separation of numerous racemic mixtures of alcohols and acids, producing optically pure compounds, such as ibuprofen, ketoprofen and naproxen. These enzymes are used to obtain poly-unsaturated fatty acids (PUFAs) from plants and animal lipids, to produce pharmaceuticals [26, 36]. Lipases are also used as additives in detergents to remove oils and fats. Therefore, they improve washing capability of detergents and enhance removal of stringent stains, preventing scaling [37]. In food and feed industry, lipases are used to modify the structure of some triglycerides for enhancing the flavor and physical and nutritional properties. They are also used in the ripening of cheese and in the production of human milk fat substitute and cocoa butter equivalents [38]. Lipases are also used in pulp and paper production to remove the hydrophobic components of wood [39]. They are also used in the synthesis of new biopolymeric materials, such as polyesters and polysaccharides, which are biodegradable and environmentally friendly [40]. One of the current applications is in transesterification reactions of plant fats for biodiesel production [41]. On the other hand, esterases are used to produce wine, fruit juices, beer, alcohol and flavoring and fragrance compounds present in cereals. In agrochemical industry these enzymes are used in the production of pesticides, insecticides, and nematicides [36]. Lipases are also used in pulp and paper to remove the hydrophobic components of wood. But they are also used in the synthesis of new biopolymeric materials, such as polyesters and polysaccharides, which are biodegradable and environmentally friendly [38–42].

## Glycosyl hydrolases (EC 3.2.1.x)

This large group of enzymes hydrolyzes glycosidic bonds between two or more carbohydrates or between carbohydrate and non-carbohydrate moieties. They degrade complex polysaccharides.

### Starch degrading enzymes

The starch-degrading enzymes use as substrate starch, one of the largest renewable carbon sources in nature. Starch is a heterogeneous polysaccharide composed of the two polymers amylose (15–25%) and amylopectin (75–85%), both are high molecular weight components. Amylose and amylopectin are composed of α-d-glucose units, linked via α-1,4-glycosidic and α-1,6-glycosidic linkages respectively, forming the insoluble linear polymer amylose and the soluble branched polymer amylopectin. Because of the complex structure of starch, starch-processing requires a combination of enzymes, which depolimerize starch into oligosaccharides and smaller sugars (endoamylases and exoamylases) and enzymes to transform starch by transferring oligoglucosidic linkages and residues, creating new bonds (debranching enzymes and glycosyl-transferases) [10, 43]. Starch-degrading enzymes also represent a large percentage of the global enzyme market.

There is a need today for thermostable enzymes as starch processing is performed at high temperatures. In addition, these enzymes should be independent from calcium and metallic ions for their stabilization and catalytic activity [43]. Thus, thermostable enzymes have the advantages of lowering the cost of sugar syrup production, consuming less energy.

### Endohydrolases (or endoamylases)

α-amylases (EC 3.2.1.1) randomly cleave α-1,4 linkages on the inner part of starch and related substrates, producing branched and linear α-anomeric oligo- and polysaccharides of different sizes. There are thermostable α-amylases from plants, fungi, animals and microbes [44]. Several of these enzymes come from hyperthermophilic archaea belonging to the genera *Pyrococcus* [45, 46], *Thermococcus* [47–49], *Desulfurococcus* [50]*, Staphylothermus* [50], *Methanococcus* [51], *and Sulfolobus* [52]. In addition, there are also α-amylases from haloalkaliphilic archaea belonging to the genera *Haloarcula* [53–55], *Halorubrum* [56], *Haloferax* [57], and *Natronococcus* [58] (see Table 5). α-amylases from haloalkaliphilic archaea are active at lower temperatures and higher pH than α-amylases from hyper/thermophilic archaea. For this reason, they are not suitable for starch industry, but they can be used in detergents for medium-temperature laundering, because of their stability in detergents and organic solvents.

**Table 5:** Starch-degrading enzymes from archaea

Enzyme	Organism	Type of enzyme	Enzyme properties			References
			Optimal temperature (°C)	Optimal pH	Thermostability	
α-Amylase	*Pyrococcus* sp. ST04	Recombinant	90–95	5.0	254 min, 75 °C	[42]
	*Pyrococcus woesei* DSM 3773	Recombinant	95	5.6	3.5 h, 110 °C	[41]
	*Thermococcus* sp.	Native	95	5.0	5 h, 90 °C	[43]
	*Thermococcus hydrothermalis*	Recombinant	75–85	5.0–5.5	Not reported	[45]
	*Methanococcus jannaschii*	Recombinant	120	5.0–8.0	50 h, 100 °C	[47]
	*Halorubrum xinjiangense*	Native	70	8.5	2 h, 70 °C	[52]
	*Haloferax mediterranei*	Native	50–60	7.0–8.0	10 h, 50 °C	[53]
β-Amylase	*Pyrococcus furiosus*	Native	110	Not reported	Not reported	[55]
Glucoamylase	*Picrophilus torridus*	Recombinant	50	5.0	8 h, 55 °C	[57]
	*Sulfolobus* *solfataricus* P2	Recombinant	90	5.5–6.0	Not reported	[58]
	*Thermoplasma acidophilum* DSM 1728	Recombinant	75	5.0	15 min, 80 °C	[59]
α-Glucosidase	*Pyrococcus furiosus*	Native	105–115	5.0–6.0	48 h, 98 °C	[62]
	*Sulfolobus tokodaii* strain 7	Recombinant	95	4.0	40.1 min, 100 °C	[65]
	*Picrophilus torridus*	Recombinant	87	5.0	120 min, 80 °C	[66]
	*Ferroplasma acidiphilum* strain Y	Recombinant	50	2.4–3.5	190 min, 50 °C	[67]
Pullulanase type II	*Thermococcus kodakarensis* KOD1	Recombinant	100	5.5–6.0	~ 2 h, 100 °C	[70]
	*Thermococcus siculi*	Recombinant	95	6.0	1 h, 100 °C	[72]
	*Desulfurococcus mucosus*	Recombinant	85	5.0	50 min, 85 °C	[73]
	*Staphylothermus marinus*	Recombinant	105	5.0	50 min, 100 °C	[75]
	*Halorubrum* sp. Strain Ha25	Native	50	7.5	212 min, 50 °C	[76]
Pullulan hydrolase type III	*Thermococcus kodakarensis*	Recombinant	95–100	3.5–4.2	45 min, 100 °C	[77]
	*Thermococcus aggregans* DSM 10597	Recombinant	95	6.5	2.5 h, 100 °C	[78]
Isoamylase	*Sulfolobus solfataricus* ATCC 35092	Recombinant	75	5.5	Not reported	[80]
Amylomaltase	*Pyrobaculum aerophilum* IM2	Recombinant	95	6.7	107 min, 95 °C	[87]
CGTase	*Pyrococcus furiosus* DSM3638	Recombinant	95	5.0	46 min, 95 °C	[88]
	*Thermococcus kodakaraensis* KOD1	Recombinant	80	5.5–6.0	20 min, 100 °C	[85]
	*Haloferax mediterranei*	Recombinant	55	7.5	~ 1 h, 50 °C (3 M NaCl)	[91]
Branching-enzyme	*Thermococcus kodakaraensis* KOD1	Recombinant	70	7.0	> 120 min, 90 °C	[92]

### Exohydrolases (or exoamylases)

β-amylases (EC 3.2.1.2) attack every alternate α-1,4-glucosidic linkage of the starch, producing the dimeric sugar β-maltose. These enzymes have been found to be distributed in higher plants, fungi, bacteria and only in one archaeon. The most thermostable β-amylase and the only one from an archaeon is PF0870, which comes from *Pyrococcus furiosus* (see Table 5). This enzyme has an optimal temperature of 110 °C, but it does not hydrolyze starch, glycogen, pullulan, or large maltooligosaccharides [59].

Glucoamylases (γ-amylases; EC 3.2.1.3) are exohydrolases that cleave α-1,4-glycosidic bonds from starch or related polysaccharides, releasing single β-d-glucose units from nonreducing ends. These enzymes also hydrolyze α-1,3- and α-1,6-glycosidic bonds in high molecular weight polysaccharides. Most of the glucoamylases reported come from fungi, but there are also in bacteria and thermoacidophilic archaea belonging to the genera *Picrophilus* [60, 61], *Sulfolobus* [62], *Thermoplasma* [60, 63], and from the methanogenic archaeon *Methanococccus* [64] (see Table 5). These archaeal glucoamylases are more thermostable than those from bacteria and fungi.

Another group of exohydrolases are α-Glucosidases (EC 3.2.1.20), which break every α-1,4- glycosidic bond from the terminal nonreducing end of starch or smaller polysaccharides produced by other starch-degrading enzymes. They prefer smaller oligosaccharides, such as maltotriose, and generate glucose [10]. These enzymes are involved in the last step of starch degradation. Most of them come from bacteria and hyperthermophilic archaea belonging to the genera *Pyrococcus* [65, 66] and *Thermococcus* [67, 68]. In addition, there are α-glucosidases from the thermoacidophilic archaea *Sulfolobus* [69] and *Picrophilus* [70], and from the acidophilic archaeon *Ferroplasma acidophilum* strain Y [71] (see Table 5).

### Starch debranching enzymes

Starch-debranching enzymes are important because of their biotechnological applications. These enzymes hydrolyze α-1,6-glycosidic bonds in amylopectin and/or glycogen and related polysaccharides. Pullulanases break down pullulan, a polysaccharide produced from starch by the fungus *Aureobasidium pullulans*. This molecule is a linear α-glucan consisting of maltotriose units joined by α-1,6-glycosidic linkages. Pullulanases are capable of hydrolyzing α-1,6 glucosidic bonds in pullulan and other branching polysaccharides, such as starch. Since the complete hydrolysis of starch can only be achieved in the presence of debranching enzymes, pullulanases are of great interest in starch industry. Based on the substrate specificity and reaction products, these enzymes are classified into three groups: pullulanases type I, pullulanases type II, and pullulan hydrolases (type I, II, and III) [72].

Pullulanases type I, exclusively hydrolyze the α -1,6 glycosidic linkages of pullulan. Are produced by *K. pneumoniae*, Bacteroides thetaiotaomicron, *Bacillus* sp. KSM-1876, *T. aquaticus,* alkaliphilic *Bacillus* sp. S-l, *Micrococcus* sp. Y-1 [73].

Pullulanases type II (amylopullulanases; EC 3.2.1.41) hydrolyze α-1,6-linkages in pullulan, producing maltotriose and also hydrolyze α-1,4-linkages in linear and branched oligosaccharides, such as amylose and amylopectin. Amylopullulanases are able to convert polysaccharides, such as amylopectin, into small sugars (e.g. glucose, maltose). These enzymes are important in starch processing industry due to their specific debranching capacity. They have been reported in bacteria and hyper/thermophilic archaea belonging to the genera *Pyrococcus* [74], *Thermococcus* [75, 76], *Desulfurococcus* [78], *Staphylothermus* [79], and in the halophilic archaeon *Halorubrum* [56] (see Table 5). Most of amylopullulanases from hyper/thermophilic archaea are active in the absence of calcium, which is a required for their industrial use.

Pullulan hydrolase type III (EC 3.2.1.x) attacks both α-1,4 and α-1,6-glucosidic linkages in pullulan, producing a mixture of maltotriose, panose, and maltose. It also degrades starch, producing mainly maltotriose and maltose [68]. This enzyme has been reported in hyperthermophilic archaea belonging to the genera *Thermococcus* [77, 82] (see Table 5).

Isoamylases (EC 3.2.1.68) are enzymes that hydrolyze α-1,6-glucosidic linkages in branched polysaccharides, such as amylopectin, glycogen, and α and β limit dextrins, producing linear malto oligosaccharides. Isoamylases are the only enzymes capable of debranching glycogen completely [79, 83]. They have been reported in plants, bacteria, and in the archaeon *Sulfolobus solfataricus* ATCC 35,092 [84] (see Table 5).

### Transferases

Transferases are enzymes that cleave an α-1,4 glucosidic bond of the donor molecule and transfer part of this molecule to a glucosidic acceptor, forming a new glucosidic bond [81].

Amylomaltases (EC 2.4.1.25) catalyze the transfer of a segment of an α-1,4-d-glucan to the reducing end of an acceptor (glucose or another α-1,4-d-glucan) [81]. These enzymes are used for producing syrups. Amylomaltases have been found in bacteria and hyperthermophilic archaea belonging to the genera *Sulfolobus* [84], *Thermococccus* [86], and *Pyrobaculum* [87] (see Table 5).

Cyclodextrin glycosyltransferases (CGTases; EC 2.4.1.19) convert starch and oligodextrins into cyclodextrins, which are six to eight α-1,4 linked glucose units with an apolar internal cavity. Most of these enzymes have been reported in bacteria and also in hyperthermophilic archaea belonging to the genera *Pyrococcus* [84], *Thermococcus* [85] as well as in *Archaeoglobus fulgidus* strain 7324 [89, 90], and from the haloalkaliphilic archaeon *Haloferax mediterranei* [91] (see Table 5).

Branching-enzymes (α-1,4-glucan 6-α-glycosyltransferase; EC 2.4.1.18) cleave α-1,4-glycosidic linkages of a linear oligo- or polysaccharide and transfer the branch to the same or another polysaccharide, creating a new α-1,6 glucosidic bond [37]. These enzymes have been reported in plants, mammals, bacteria, fungi, and in the archaeon *Thermococcus kodakaraensis* KOD1 [88] (see Table 5).

### Biotechnological applications of starch-degrading enzymes

In food and feed industry starch-converting enzymes are used to produce valuable products (glucose, fructose, and maltose) from starch. It is possible to produce starch-based materials with gelatin-like characteristics and defined linear dextrines as texturizers, aroma stabilizers, and prebiotics [37]. Pullulanases and amylopullulanases are used for the production of glucose, maltose, and fructose as food sweeteners. These enzymes are also used for the production of high-glucose, high-fructose, and high-maltose syrups (manufacturing of high-quality candy and ice cream). In baking industry, pullulanases are used to improve texture, volume, and flavor of bakery products [68]. Amylomaltases can produce cycloamylose and thermoreversible starch gel, which can be used as a substitute of gelatin [81]. They are also used to produce syrups of isomalto-oligosaccharides with low sweetness and viscosity. α-amylases, branching and debranching enzymes and β-amylases can act as anti-staling agents, preventing undesirable changes in bread [37]. In pharmaceutical industry and human health, pullulanases can be used for the production of maltose, which can replace d-glucose in the intravenous feeding [68]. These enzymes are also used for the production of branched cyclodextrins. Due to their apolar interior, cyclodextrins can be used as hosts for pharmaceutical important molecules (e.g. proteins) that are solubilized and stabilized. On the other hand, pullulanases debranching are used for the preparation of slowly digestible starch, which correlates with low glycemic levels [10]. Alkaline pullulanases and α-amylases are used as additives in dishwashing and laundry detergents to remove starches under alkaline conditions [68]. In biofuel production α-amylases, glucoamylases, pullulanases and amylopullulanases can be used for degrading starch-containing crops (e.g. wheat, corn, barley) and produce ethanol [10].

### Cellulases

Cellulose is the most abundant polymer on earth. This polysaccharide is a structural component of the cell wall of green plants and consists of up to 15,000 glucose units linked by β-1,4-glycosidic bonds. It has a high affinity to water, but it is completely insoluble in it because of its heterogeneous structure, which consists of both amorphous and highly ordered crystalline regions. Cellulases hydrolyze β-1,4 linkages in cellulose and based on their amino acid sequences and crystal structures they have been classified into three types: endoglucanases, exoglucanases, and β-glucosidases. Due to the complex structure of cellulose, it is necessary the combination of these enzymes for the complete hydrolysis of it into glucose. Cellulose is typically embedded in a network of hemicellulose and lignin, for this reason it requires an alkaline pretreatment at high temperatures to become accessible to enzymatic hydrolysis. So, cellulose industry needs thermostable cellulases, which in addition must be active at high pH [37, 81].

The endoglucanases (Cellulases; EC 3.2.1.4) hydrolyze β-1,4 bonds of cellulose in a random manner, generating oligosaccharides, such as cellobiose and glucose. These enzymes have been reported in bacteria, fungi, plants, animals, and in the hyperthermophilic archaea belonging to the genera *Pyrococcus* [93–96], *Ignisphaera* [97], *Metallosphaera* [98], *Thermoproteus* [99]. These enzymes are also been reported in the thermoacidophilic archaea *Acidilobus saccharovorans* [100], *Sulfolobus solfataricus* [101], and in the haloalkaliphilic archaeon *Haloarcula* [102–104] (see Table 6). Endoglucanases from *Ignisphaera aggregans*, *Metallosphaera cuprina*, *Thermoproteus uzoniensis*, and *Acidilobus saccharovorans* have been identified but they are not characterized.

**Table 6:** Cellulose-degrading enzymes from archaea

Enzyme	Organism	Type of enzyme	Enzyme properties			References
			Optimal temperature	Optimal pH	Thermostability	
Endoglucanase	*Pyrococcus horikoshii*	Recombinant	> 97 °C	5.6	3 h, 97 °C	[94]
	*Sulfolobus solfataricus* P2	Recombinant	80 °C	1.8	8 h, 80 °C	[101]
	*Haloarcula* sp. LLSG7	Native	50 °C (20% NaCl)	8.0 (20% NaCl)	72 h, < 70 °C	[102]
*β*-Glucosidase	*Pyrococcus furiosus*	Recombinant	95 °C	5.5	88 h, 95 °C	[103, 104]
	*Sulfolobus solfataricus*	Recombinant	90 °C	5.5	18 h, 90 °C	[105]
	*Thermofilum pendens*	Recombinant	90 °C	3.5	60 min, 95 °C	[107]

β-glucosidases (Cellobiases; EC 3.2.1.21) hydrolyze soluble cellodextrins and cellobiose, releasing β-d-glucose. These enzymes have been reported in bacteria, fungi, plants, animals, and archaea belonging to the genera *Pyrococcus* [94, 103, 104], and the thermoacidophilic archaea *Sulfolobus* [104–106] and *Thermofilum* [107] (see Table 6).

### Biotechnological applications of cellulose-degrading enzymes

In pulp and paper industry, mixtures of endoglucanases reduce the fiber coarseness. Endoglucanases decrease the pulp viscosity and cellulases enhance the bleachability of softwood kraft pulp. Cellulases and xylanases release the ink from the fiber surface, improve fiber brightness and strength properties. In food and feed industry, cellulases are used for the improvement of juice yield, pretreatment of cellulose biomass and forage crops to improve nutritional quality. These enzymes are also employed in the color extractions of juices and releasement of antioxidants from fruit pomace. β-glucosidases improve texture, flavor, aroma of fruits and vegetables, they control bitterness of citrus fruits, and are used as additives to hydrolyze nonstarch polysaccharides [10, 108]. In biofuel production cellulases are used to increase the yield of saccharification of agricultural and industrial waste for bioethanol production. These enzymes convert cellulosic materials into useful and valuable products, such as ethanol, solvents, and organic acids. They improve nutritional quality of animal feed and facilitate their digestion and absorption. Textile industry also uses these enzymes for biostoning of jeans and biopolishing of cotton. Endoglucanases improve softness and water absorbance property of fibers and provide a cleaner surface structure. Cellulases remove short fibers, create a smooth appearance, and improve color brightness. In home care industry particularly in detergents, cellulases are used as additives causing color brightening and softening of fibers and removing rough protuberances in cotton fabrics [37]. For wine and beer industry glucanases can improve quality, fermentation, and yields of beers and wines. These enzymes together with β-glucosidases improve color extraction, maceration, clarification, filtration, stability and aroma of wines. In agricultural industry, preparations based on cellulases are used to control plant disease because they can degrade the cell wall of phytopathogens [108].

### Xylanases

The starting material to produce paper is wood, which is composed of cellulose (40–45%), hemicellulose (20–30%), and lignin (15–25%). Xylan, the principal component of hemicellulose, is a heterogeneous molecule with a main chain composed of xylose residues linked by β-1,4-glycosidic bonds [10]. Xylanases are present in bacteria, fungi, and archaea. The steps in the paper production are carried out at elevated temperatures, so this industry requires thermostable xylan-degranding enzymes [109].

The endo-β-1,4-xylanases (xylanase; EC 3.2.1.8**)** are the most predominant enzymes. They cleave β-1,4-xylosidic linkages in xylans [85]. These enzymes have been reported in the halophilic archaeon *Halorhabdus utahensis* [110] and in the hyperthermophilic archaeon *Pyrodictium abyssi* [111] (see Table 7). On the other hand, β-1,4-Xylosidases (EC 3.2.1.37) hydrolyze β-1,4-xylans and disaccharides, such as xylobiose, generating D-xylose [37]. These enzymes have been reported in the halophilic archaeon *Halorhabdus utahensis* [110] and in the thermoacidophilic archaeon *Sulfolobus solfataricus* [112] (see Table 7).

**Table 7:** Xylan- and chitin-degrading enzymes from archaea

Enzyme	Organism	Type of enzyme	Enzyme properties			References
			Optimal temperature (°C)	Optimal pH	Thermostability	
Endo-β-1,4-xylanase	*Pyrodictium abyssi*	Native	110	5.5	100 min, 105 °C	[111]
β-1,4-Xylosidase	*Sulfolobus solfataricus* strain MT4	Native	90	7.0	47 min, 100 °C	[112]
Chitinases	*Halobacterium salinarum* CECT 395	Recombinant	40	7.3	40 min, 45 °C	[113]
	*Sulfolobus tokodaii*	Recombinant	70	2.5	–	[119]
	*Thermococcus chitonophagus*	Native	70	7.0	1 h, 120 °C	[121]

### Biotechnological applications of xylanases

In pulp and paper industry xylanases are used in bleaching of cellulose pulp as an alternative to chlorine bleaching. The treatment with these enzymes makes the pulp more permeable to subsequent extraction of residual brown lignin from fibers, because they degrade the xylan network that traps the residual lignin. In food and feed industry xylanases in conjunction with cellulases, and amylases improve yield and clarification of fruit juices. These enzymes increase aromas, essential oils, pigments, etc. of fruits and vegetables. Xylanases are also used as ingredients during bread preparations to improve its quality. In animal feed, these enzymes along with cellulases, proteases, and lipases are used to digest raw material, reducing viscosity, which improve the digestion of nutrients [110–112]. In pharmaceutical industry and human health, xylanases in conjunction with proteases are used as dietary supplements or to treat poor digestion. On the other hand, hydrolytic products of xylan are used as low-calorie sweeteners [114].

### Chitinases

Chitin is the second most abundant polysaccharide, after cellulose, present in fungal cell walls, insect exoesqueletons, and shells of crustacea. Chitin, a linear β-1,4 homopolymer of *N*-acetyl-d-glucosamine (GlcNAc) residues, is crystalline, highly hydrophobic, and insoluble in water and organic solvents. This polysaccharide is nontoxic, antimicrobial, and biodegradable polymer. It is used for the production of oligosaccharides as biologically active substances [115]. Chitinases have been reported in bacteria, fungi, plants, insects, mammals, and archaea belonging to the genera *Haloferax* [116], *Halobacterium* [117], *Pyrococcus* [118], *Sulfolobus* [119], *Thermococcus* [120, 121] (see Table 7).

### Biotechnological applications of chitinases

Chitinases are used for the preparation of pharmaceutical important chitooligosaccharides with anti-tumor activity and *N*-acetyl-d-glucosamine, which is an anti-inflammatory drug used in the treatment of osteoarthritis, ulcerative colitis and other gastrointestinal inflammation disorders. Degradation products of chitin are used in drug delivery, wound healing, anti-fungal creams and lotions, production of artificial skin, surgical stitches and dietary fiber. These chitin derivatives are non-toxic, non-allergic, biodegradable, and biocompatible. In agricultural industry chitinases are used to control fungal phytopathogens and harmful insects, degrading their chitin coats. For bioremediation they are used in the treatment of chitinous waste to fertilizer [122].

## DNA-processing enzymes

DNA polymerases and DNA ligases are enzymes widely used in molecular biology to perform the polymerase chain reaction (PCR) and analytical methods, respectively [123].

DNA polymerases (EC 2.7.7.7) are key enzymes in DNA replication in all life forms. They synthesize a new DNA strand according to the template DNA, adding a deoxyribonucleotide 5′-triphosphate onto the growing 3′-OH end of a primer strand in the presence of Mg^2+^ ions. *Taq* polymerase was the first thermostable DNA polymerase applied in PCR but it has not 3′–5′ proofreading exonuclease activity, as a result, this enzyme is unable to excise mismatches. So, when high fidelity is required to reduce the error rate, the best choice is an archaeal DNA polymerase. These DNA polymerases have 3′–5′ proofreading activity, an error rate tenfold lower than that of *Taq* polymerase and are more thermostable, but are slower. The most commonly used DNA polymerases are from the archaea belonging to the genera *Pyrococcus* (*Pfu*, *Pwo*, Deep Vent™, Platinum^*®*^ *Pfx*) and *Thermococcus* (KOD1, Tli, 9°N-7) [123–125] (see Table 8).

**Table 8:** DNA polymerases from archaea. Adapted from [120]

DNA polymerases	Organism	Half-life	References
Pfu	*Pyrococcus furiosus*	95% activity after 1 h, 95 °C	[125]
Deep Vent™	*Pyrococcus* species GB-D	23 h, 95 °C	[124]
Pwo	*Pyrococcus woesei*	2 h, 100 °C	[126]
Pab (Isis™)	*Pyrococcus abyssi*	5 h, 100 °C	[127]
Tli (Vent™)	*Thermococcus litoralis*	6.7 h, 95 °C	[125]
KOD1	*Thermococcus kodakaraensis*	12 h, 95 °C/3 h, 100 °C	[128]
Tfu	*Thermococcus fumiculans*	3.3 h, 95 °C/2 h, 100 °C	[129]
TNA1_pol	*Thermococcus* sp. NA1	12.5 h, 95 °C/3.5 h, 100 °C	[130]

On the other hand, DNA ligases (ATP-dependent DNA ligases, EC 6.5.1.1 and NAD+ -dependent DNA ligases, EC 6.5.1.2) are ubiquitous enzymes that ligate breaks in DNA. The first thermostable ligase was discovered in the bacterium *Thermus thermophilus* HB8. Most of these enzymes come from thermophilic bacteria, but there are also of them from the hyper/thermophilic archaea *Pyrococcus* [131–133], *Thermococcus* [133–136], *Hyperthermus butylicus* [137], *Methanocaldococcus jannaschii* [138], *Methanobacterium thermoautotrophicum* [139], *Sulfophobococcus zilligii* [140], *Aeropyrum pernix K1* [141], *Archaeoglobus fulgidus* [142], and *Sulfolobus* [143] (see Table 9). Unlike bacterial DNA ligases, these enzymes require ATP as a cofactor.

**Table 9:** DNA ligases from archaea

Organism	Half-life	References
*Pyrococcus abyssi*	60 min, 90 °C	[132]
*Thermococcus fumiculans*	3 h, 80 °C	[136]
*Hyperthermus butylicus*	2.3 h, 94 °C/1.7 h, 99 °C	[137]
*Methanocaldococcus jannaschii*	20 min, 90 °C	[138]
*Aeropyrum pernix* K1	25 min, 110 °C	[141]

### Biotechnological applications of DNA polymerases and DNA ligases

Thermostable DNA polymerases are used in DNA amplification, sequencing or labelling. Due to the high fidelity of archaeal DNA polymerases, they are used for reducing amplification errors in PCR products. On the other hand, thermostable DNA ligases are used in the construction of sequencing primers and as LDR/LCR enzymes because of their catalytic activity for nick-joining reaction at high temperatures (90–100 °C). LDR/LCR is a technique for detecting a single-base mutation in the DNA strand and it is used for the diagnosis of genetic diseases [10].

## Nitrile-degrading enzymes

Nitriles are organic compounds that contain a cyano group (−C≡N) as part of their chemical structure. They are important chemical building blocks for the synthesis of intermediates in fine chemicals and pharmaceuticals. These reactions are carried out at elevated temperatures. So, pharmaceutical industry requires thermostable nitrile-degrading enzymes (amidases and nitrilases) [1, 144].

Amidases (EC 3.5.1.4) catalyze the conversion of amides to the corresponding carboxylic acids and ammonia. These enzymes are enantioselective and have a diverse substrate spectrum. Most of them do not require metal ions to be active. There are amidases that hydrolyze aliphatic substrates (aliphatic amidases) and those that hydrolyze cyclic or aromatic amides (aromatic amidases). These enzymes have been reported in bacteria and in hyper/thermophilic archaea belonging to the genera *Pyrococcus* [146] and *Sulfolobus* [147–149] (See Table 10).

**Table 10:** Nitrile-degrading enzymes from archaea

Enzyme	Organism	Type of enzyme	Enzyme properties			References
			Optimal temperature (°C)	Optimal pH	Thermostability	
Amidase	*Pyrodoccus yayanosii*	Recombinant	85	6.0	110 min, 80 °C	[146]
	*Sulfolobus tokodaii s*train 7	Recombinant	75	7.0–8.0	–	[147]
Nitrilase	*Pyrococcus abyssi*	Recombinant	80	7.4	6 h, 90 °C	[149]
Nitrilase	New *Pyrococcus* sp. from Antarctica	Recombinant	90	7.0	8 h, 90 °C losses 50% of activity	[145]

On the other hand, nitrilases (EC 3.5.5.1) hydrolyze in one step nitriles to their corresponding carboxylic acid and ammonia. They are regio-, chemo-, and enantioselective, have a wide substrate spectrum, and do not require metal ions to be active. However, most of nitrilases have a poor thermostability. Based on the substrate specificity these enzymes are classified as aliphatic nitrilases (high affinity for aliphatic nitriles), aromatic nitrilases (high affinity for aromatic and heterocyclic nitriles), and arylacetonitrilases (high affinity for arylacetonitriles). Most of these enzymes come from bacteria, but they are also present in plants, fungi and archaea. Only one recombinant nitrilase have been reported from the hyperthermophilic archaeon *Pyrococcus abyssi.* This enzyme is an aliphatic nitrilase with high thermostability. Nevertheless, it does not hydrolyze aromatic nitriles, which are widely used in fine chemical and pharmaceutical industries [149].

In our laboratory we have isolated a nitrilase from a novel Antarctic *Pyrococcus* sp. recently isolated from the Antarctic Peninsula, Deception Island. This microorganism was isolated from an environmental sample and it was able to grow in the presence of aromatic nitriles at temperatures above 80 °C. The gene encoding the nitrilase enzyme was identified from its genome and subsequently was cloned and overexpressed in *E. coli.* The recombinant nitrilase showed activity at elevated temperatures towards aromatic and aliphatic nitriles, although it hydrolyzes preferentially aromatic compounds. The specific catalytic properties of this enzyme make it a potential candidate as biocatalyst for pharmaceutical industry [145]. Currently, the complete biochemical characterization and thermostability studies of this enzyme are taking place.

### Biotechnological applications of nitrile-degrading enzymes

Amidases are used to produce optically pure compounds in pharmaceutical industry. In food industry these enzymes are used to produce glutamic acid and aspartic acid (which contribute to the tastes “umami” and “sour”) and to produce fermented condiments such as soy sauce. Another application of these enzymes is in waste water treatment [1, 150].

On the other hand, nitrilases are used for the production of active pharmaceutical ingredients (API) and drug intermediates. This includes the synthesis of common and valuable pharmaceuticals, such as non-steroidal anti-inflammatory drugs (Ibuprofen, Ketoprofen, Naproxen), antidepressants (Nialamide, Gabapentin), analgesics (Pregabalin), anticholesterol drugs (Atorvastatin, Resuvastatin), anxiolytic and anticonvulsive drugs (Pregabalin), anti-tumor agents (Taxol), antibiotics (penicillins and semisynthetic cephalosporins), anti-allergic drugs (Terfenadine), drug delivery material (polyglycolic acid), and dermatological products (glycolic acid and mandelic acid, which are used as additives in facial moisturizers). In chemical industry nitrilases are used for the production of acrylamide, which is used in molecular biology (SDS-PAGE), paper making, permanent press fabrics, and ore processing. These enzymes are used to synthesize plastics, surface coatings, adhesives, synthetic rubbers, cosmetics, perfumes, household cleaners, automotive oil additives, pesticides, and herbicides. Additionally, nitrilases are used to produce glycine, which is a raw material for synthesizing detergents. They are used for synthesizing nicotinic acid (vitamin B3), which is used in the production of feedstuff additive. Since nitrilases are able to degrade nitriles, they can also be used for the treatment of contaminated water and soil [144, 151].

This review has summarized the main industrial enzymes from archaea, which play key roles in processes carried out in food, beverages, paper, textile, detergent, and pharmaceutic industries. However, there are also archaeal enzymes with specific applications which have been patented. Some of them are summarized in Table 11.

**Table 11:** Patents related with archaeal enzymes

Enzyme	Origin	Application	Year	References
Adenylate kinase	*Sulfolobus acidocaldarius*	Biological indicator for validation of procedures to inactivate transmissable spongiform encephalopathy (TSE) agents	2015 (grant)	[152]
β-glycosidase	*Pyrococcus furiosus*	Production of ginseng compounds for medical applications	2012 (grant)	[153]
DNA polymerases	*Pyrococcus* and *Thermococcus*	Recombinant DNA technologies	2013 (grant)	[154]
Endoglucanase	*Pyrococcus horikoshii*	Degradation of natural crystalline cellulose in textile industry	2015 (application)	[155]
Glucosidases	*Thermococcus*, *Staphylothermus* and *Pyrococcus*	Food processing, pharmaceutical, textile, detergent, and baking industries	2014 (application)	[156]

From the archaeal enzymes currently described and used in biotechnological applications several of these enzymes belong or have been isolated from species which recently have been described and found for the first time in Antarctica. Among them *Micrococcus, Thermoccocus, Pyroccocus.*

The enzyme nitrilase has been recently isolated and characterized from a novel *Pyroccocus* specie found in Antarctica by our group and it is currently being fully identified.

## Conclusions

Archaeal extremozymes have demonstrated to be excellent biocatalysts for industrial applications due to their stability at high temperatures, extremes of pH, organic solvents, high salt concentration, and enantio selectivity. Due to these properties, archaeal biocatalysts can be used in a wide range of biotechnological applications. They can improve the processing of starch, cellulose, chitin, and xylan and they also allow the production of enantiomerically pure drugs of common use. Global enzyme market includes many industrial sectors and for this reason it requires large amounts of different enzymes. The best option to fulfill these requirements is the use of recombinant enzymes, which are produced in large-scale and can be easily purified. Nevertheless, nowadays there is still a need for more novel enzymes that can be generated in a recombinant manner particularly from archaea.

Recent findings of interesting archaeal species in Antarctica containing novel enzymes for potential industrial applications, makes Antarctica an interesting source of new archaeal and other type of microorganisms which contain more stable and active enzymes.

The task today and for the future is to generate better molecular tools for overexpression of some of these novel enzymes which are poorly expressed in the currently available molecular tools. The search for more enzymes and efficient improvements through modern technologies, such as site-directed mutagenesis, directed evolution, rational design, and metabolic engineering in order to generate the new generation of industrial biocatalysts is still needed.

## Abbreviations

MPa: mega pascale
kGy: kilo gray
Gy: gray
DNA: deoxyribonucleic acid
PCR: polymerase chain reaction
PUFA: poly unsaturated fatty acids
LCR: ligase chain reaction
LDR: ligase detection reaction
SDS-PAGE: sodium dodecyl sulfate polyacrylamide gel electrophoresis
TSE: transmissible spongiform encelopathy
